# Fermentative hydrogen production from agroindustrial lignocellulosic substrates

**DOI:** 10.1590/S1517-838246220140111

**Published:** 2015-06-01

**Authors:** Valeria Reginatto, Regina Vasconcellos Antônio

**Affiliations:** 1 Universidade de São Paulo, Faculdade de Filosofia, Ciências e Letras de Ribeirão Preto, Universidade de São Paulo, Ribeirão Preto, SP, Brasil, Departamento de Química, Faculdade de Filosofia, Ciências e Letras de Ribeirão Preto, Universidade de São Paulo, Ribeirão Preto, SP, Brazil.; 2 Universidade Federal de Santa Catarina, Universidade Federal de Santa Catarina, Araranguá, SC, Brasil, Universidade Federal de Santa Catarina, Araranguá, SC, Brazil.

**Keywords:** fermentation, hydrogen, lignocellulosic substrates, pretreatment, inhibitors

## Abstract

To achieve economically competitive biological hydrogen production, it is crucial to consider inexpensive materials such as lignocellulosic substrate residues derived from agroindustrial activities. It is possible to use (1) lignocellulosic materials without any type of pretreatment, (2) lignocellulosic materials after a pretreatment step, and (3) lignocellulosic materials hydrolysates originating from a pretreatment step followed by enzymatic hydrolysis. According to the current literature data on fermentative H_2_ production presented in this review, thermophilic conditions produce H_2_ in yields approximately 75% higher than those obtained in mesophilic conditions using untreated lignocellulosic substrates. The average H_2_ production from pretreated material is 3.17 ± 1.79 mmol of H_2_/g of substrate, which is approximately 50% higher compared with the average yield achieved using untreated materials (2.17 ± 1.84 mmol of H_2_/g of substrate). Biological pretreatment affords the highest average yield 4.54 ± 1.78 mmol of H_2_/g of substrate compared with the acid and basic pretreatment - average yields of 2.94 ± 1.85 and 2.41 ± 1.52 mmol of H_2_/g of substrate, respectively. The average H_2_ yield from hydrolysates, obtained from a pretreatment step and enzymatic hydrolysis (3.78 ± 1.92 mmol of H_2_/g), was lower compared with the yield of substrates pretreated by biological methods only, demonstrating that it is important to avoid the formation of inhibitors generated by chemical pretreatments. Based on this review, exploring other microorganisms and optimizing the pretreatment and hydrolysis conditions can make the use of lignocellulosic substrates a sustainable way to produce H_2_.

## Introduction

H_2_ is a promising fuel: it is carbon-free and its combustion produces only water (Wang and Wan, 2009). Although H_2_ constitutes a clean fuel, currently available methods leading to its production, such as methane reforming and partial oil and coal oxidation, demand fossil fuels and a high amount of energy (Chaubey *et al.*, 2013). Biological approaches that produce H_2_ offer several advantages over current physicochemical methods: they occur at ambient temperature and pressure, and they use renewable raw materials as substrates (Li and Fang, 2007; Li *et al.*, 2012).

A number of microbes belonging to a wide variety of bacterial groups can perform fermentative H_2_ production, also called dark fermentation because it does not require light. The strict anaerobe *Clostridium* spp. and facultative anaerobes from the family Enterobacteriaceae are the most often cited H_2_-producing bacteria (Seol *et al.*, 2008, Elsharnouby *et al.*, 2013).

Mixed cultures that usually originate from an anaerobic environment, such as the sludge from anaerobic biodigestors, have also found application in H_2_-producing processes. They resist the fluctuations typical of the fermentation process, consume a broader range of complex substrates, and can operate in a non-sterile environment (Valdez-Vazquez and Poggi-Varaldo, 2009, Kothari *et al.*, 2012, Show *et al.*, 2012; Rafrafi *et al.*, 2013).

However, it is the choice of substrate for fermentative H_2_ production that determines the feasibility of the process. The substrate should (1) be carbohydrate-rich, (2) originate from renewable resources, (3) suffice for fermentation, and (4) promote energetically favorable energy recovery. In addition, any necessary pretreatment should be inexpensive (Wang and Wan, 2009; Chaubey *et al.*, 2013). In this context, several investigators have turned to lignocellulosic materials to produce H_2_ (Kapdan and Kargi, 2006; Ren *et al.*, 2009; Lin *et al.*, 2012). According to Kotay and Das (2008), if the use of these resources is appropriately controlled, they will become a major source of energy in the future. Unfortunately, these residues have a complex chemical structure and often call for previous treatment and/or hydrolysis to serve as substrate for biological H_2_ production. Such pretreatment and/or hydrolysis could not only alter the physicochemical features of the waste, making carbohydrates available for fermentation, but also afford byproducts that negatively interfere in fermentative H_2_ production.

This review compares the yields of fermentative H_2_ production from (1) different agroindustrial lignocellulosic substrates without any chemical or biological pretreatment (2) lignocellulosic materials after a pretreatment step and (3) hydrolysates of lignocellulosic materials originating from a pretreatment step followed by enzymatic hydrolysis. The comparison of these results will show how the pretreatment and hydrolysis of lignocellulosic substrates affect fermentative H_2_ production. In addition, this review will present the microorganisms involved in H_2_ production from those materials.

## Lignocellulosic Materials as Substrate for Fermentative H_2_ Production

Lignocellulosic materials are the most abundant residues derived from agroindustrial activities; therefore, they can potentially become a significant source of renewable H_2_ (Saratale *et al.*, 2008; Levin *et al.*, 2009; Ren *et al.*, 2009; Cheng *et al.*, 2011; Hay *et al.*, 2013). Agricultural residues from harvested crops are the cheapest and the most abundant readily available lignocellulosic organic waste; they include straw, stover, peelings, cobs, stalks, and bagasse (Guo *et al.*, 2010a; Cheng *et al.*, 2011; Li *et al.*, 2012). All these residues can undergo biological transformations to varying degrees, as well as conversion to hydrogen (Guo *et al.*, 2010a).

Researchers have investigated several agroindustrial wastes for H_2_ production. Cornstalk (Cao *et al.*, 2009; Cao *et al.*, 2012; Cheng *et al.*, 2012; Song *et al.*, 2012; Zhao *et al.*, 2013), wheat straw (Fan *et al.*, 2006; Kaparaju *et al.*, 2009; Kongjan and Angelidaki, 2010; Nasirian *et al.*, 2011, Quemeneur *et al.*, 2012a) and sugarcane bagasse (Pattra *et al.*, 2008; Chairattanamanokorn *et al.*, 2009; Fangkum and Reungsang, 2011) are the most cited in the literature.

Lignocellulosic materials consist primarily of cellulose, hemicelluloses, and lignin. Thus, the main products of the enzymatic, chemical, or thermochemical hydrolysis of lignocellulosic materials are hexoses, mainly glucose, and pentose sugars, mainly xylose.

In addition to H_2_, the anaerobic digestion of glucose by strict anaerobes or facultative microorganisms yields different final products. Depending on the bacterial species, pH, and H_2_ partial pressure, the fermentation of glucose can result in H_2_, CO_2_, acetate and/or butyrate (Eqs. 1 and 2). Theoretically, when the final product is acetate only, 4 mol of H_2_/mol of glucose can emerge (Eq. 1). However, if the final product is butyrate, only 2 mol of H_2_/mol of glucose arises (Eq. 2).

Xylose is the major pentose derived from the hydrolysis of hemicelluloses, which in turn constitutes approximately 20 to 30% of plant biomass. It can be used for the growth and energy production of numerous microorganisms. The use of xylose as a substrate for ethanol production has been extensively studied (Sun and Cheng, 2002; Lin and Tanaka 2006; Sarks *et al.*, 2014). However, only recently has attention been given to H_2_ production from xylose fermentation. Theoretically, similarly to glucose fermentation, xylose fermentation can produce 3.33 mol H_2_/mol xylose when acetate is the fermentation product (Eq. 3). When butyrate is the fermentation product, 1.66 mol of H_2_/mol of xylose will emerge (Eq. 4) (Martin del Campo *et al.*, 2013).

(1) $${\text{C}}_{6}{\text{H}}_{12}{\text{O}}_{6}+2{\text{H}}_{2}\text{O}\to 2{\text{CH}}_{3}{\text{COO}}^{-}+2{\text{CO}}_{2}+4{\text{H}}_{2}$$

(2) $${\text{C}}_{6}{\text{H}}_{12}{\text{O}}_{6}\to {\text{CH}}_{3}{\text{CH}}_{2}{\text{CH}}_{2}{\text{COO}}^{-}+2{\text{CO}}_{2}+2{\text{H}}_{2}$$

(3) $${\text{C}}_{5}{\text{H}}_{10}{\text{O}}_{5}+1.66{\text{H}}_{2}\text{O}\to 1.66{\text{CH}}_{3}{\text{COO}}^{-}+1.66{\text{CO}}_{2}+3.33{\text{H}}_{2}$$

(4) $${\text{C}}_{5}{\text{H}}_{10}{\text{O}}_{5}+1.66{\text{H}}_{2}\text{O}\to 1.66{\text{CH}}_{3}{\text{CH}}_{2}{\text{CH}}_{2}{\text{COO}}^{-}+1.66{\text{CO}}_{2}+1.66{\text{H}}_{2}$$

**Figure 1:**
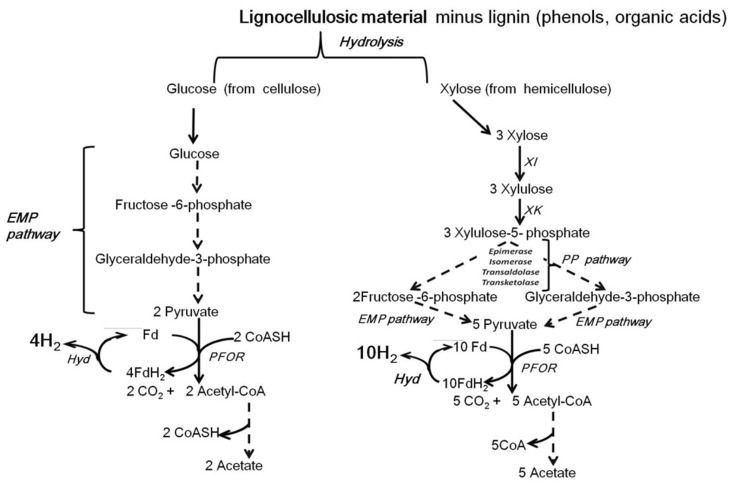
Schematic view of the major metabolic pathways that lead to the production of H_2_, CO_2_, and acetate from the carbohydrate components obtained from the hydrolysis of lignocellulosic materials. EMP, Embden-Meyerhoff-Parma; Fd, oxidized ferredoxin; FdH_2_, reduced ferredoxin; Hyd, hydrogenase; PFOR, pyruvate: ferredoxin oxyreductase; PP, pentose phosphate; XI, xylose isomerase; XK, xylulokinase. The dashed arrows indicate multisteps of a metabolic pathway.

Figure 1 shows the main steps of the metabolic pathways and enzymes leading to H_2_ production throughout glucose and xylose fermentation performed by anaerobic microorganisms. The figure shows that the enzyme xylose isomerase (XI) catalyzes the isomerization of xylose to xylulose. The latter is then phosphorylated by xylulokinase (XK), to afford xylulose-5-phosphate, one of the intermediates of the pentose phosphate (PP) pathway. Through the activities of epimerase, isomerase, transketolases, and transaldolases, enzymes of the PP pathway, xylulose-5-phosphate is converted to fructose-6-phosphate and glyceraldehyde-3-phosphate. Both of these compounds are intermediates of the EMP pathway, through which they undergo conversion to pyruvate. The supposed activities of pyruvate, ferredoxin oxyreductase (PFOR) and ferredoxin-dependent hydrogenase (Hyd) will produce H_2_, CO_2_, and acetate.

According to the Figure 1, glucose is converted to pyruvate, from which H_2_, CO_2_, and acetate are produced, as outlined above. It is noteworthy that for both carbohydrates, the consumption of reducing power to generate butyrate instead of acetate reduces the H_2_ yield.

To produce H_2_ by fermentation, it is possible to use (1) lignocellulosic materials without any chemical or biological pretreatment, (2) lignocellulosic materials after a pretreatment step, or (3) hydrolysates of lignocellulosic materials that normally originate after a pretreatment step followed by enzymatic hydrolysis. Another approach is to conduct simultaneous saccharification and fermentation (SSF), which consists in adding a hydrolytic enzyme(s) or microorganisms to a fermentation vessel (Quemeneur *et al.*, 2012a).

## Pretreatment of Lignocellulosic Materials for Fermentative H_2_ Production

The complex nature of lignocellulosic substrates may adversely affect their biodegradability. Therefore, prehydrolysis, often referred to as pretreatment, is required to alter the structure of lignocellulosic biomass to make the sugars available for fermentation (Ren *et al.*, 2009, Levin *et al.*, 2009). Carbohydrate polymers (cellulose and hemicellulose) and lignin are the main components of lignocellulosic materials (Rezende *et al.*, 2011; Mood *et al.*, 2013). Agricultural residues such as wheat straw, corn stalk, sugarcane bagasse, and rice straw contain approximately 32–47% cellulose, 19–27% hemicellulose, and 5–24% lignin (Sun and Cheng, 2002). Although hemicellulose and lignin are minor components, they protect cellulose. Hence, it is necessary to hydrolyze these components, to efficiently use the cellulose (Mosier *et al.*, 2005; Rezende *et al.*, 2011). Thus, appropriate pretreatment steps reduce the cellulose crystallinity and/or polymerization degree and selectively remove hemicellulose and lignin to make carbohydrates from lignocellulosic materials accessible for enzymatic hydrolysis (Mood *et al.*, 2013; Monlau *et al.*, 2013a).

The main pretreatment methods rely on mechanical, physical, chemical, and biological techniques or a combination thereof (Alvira *et al.*, 2010; Guo *et al.*, 2010b; Ogeda and Petri, 2010). These methods serve to prepare lignocellulosic materials for bioethanol production mainly, but most of them also find application in fermentative H_2_ production (Guo *et al.*, 2010a; Mood *et al.*, 2013; Monlau *et al.*, 2013a).

Physicochemical pretreatment includes steam explosion, steam explosion with ammonium, use of organic solvents and supercritical fluids, and use of diluted acids and/or bases (Mosier *et al.*, 2005; Vargas Betancur and Pereira Jr, 2010; Monlau *et al.*, 2013b). Biological pretreatment relies on the ability of fungi and bacteria to produce enzymes such as lignin peroxidase and laccase, and hemicellulase, which help to remove lignin and hemicellulose from the lignocellulosic matrix, respectively (Ogeda and Petri, 2010).

Various methods for pretreating lignocellulosic material exist; however, it is essential to select a method that minimizes carbohydrate degradation and avoids the formation of inhibitory compounds that are toxic to fermentative microorganisms (Alriksson *et al.*, 2011; Rezende *et al.*, 2011; Jonsson *et al.*, 2013). Pretreatment at high temperatures rapidly degrades hemicellulose pentoses and to a lesser extent hexoses, producing acetic acid and furfurals, which constitute potential fermentation inhibitors (Alriksson *et al.*, 2011; Jonsson *et al.*, 2013).

**Figure 2:**
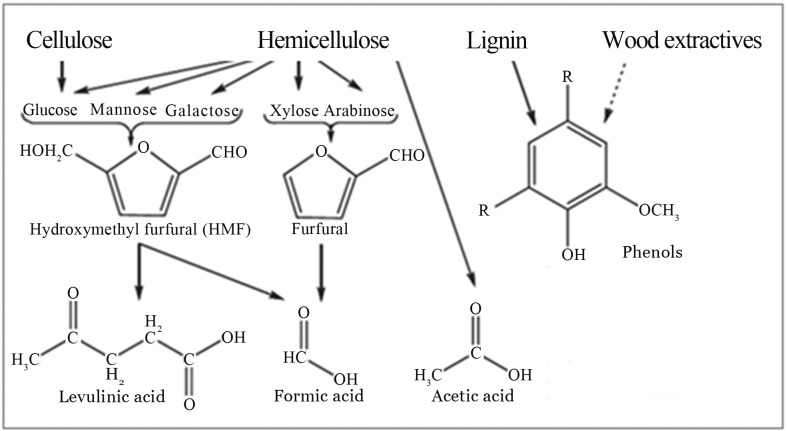
Products and subproducts from the pretreatment of lignocellulosic materials (modified from Jonsson *et al.*, 2013).

Figure 2 shows the main carbohydrate degradation products from hemicelluloses and cellulose hydrolysis, *i.e.*, xylose and glucose, as well as furfural, hydroxymethylfurfural (HMF), and organic acids, such as formic and acetic acid (Palmqvist and Hahn-Hagerdal, 2000; Jonsson *et al.*, 2013).

Furfural originates from pentose dehydration; its concentration in the liquid phase increases with rising pretreatment temperature, acid concentration, or pretreatment time (Chen *et al.*, 2013). Furfural may react further, to yield formic acid, or it may polymerize. Hydroxymethylfurfural (HMF) stems from the dehydration of hexoses such as glucose; it can further react to yield levulinic and formic acid (Palmqvist and Hahn-Hagerdal, 2000; Chen *et al.*, 2013; Jonsson *et al.*, 2013). These inhibitors may interfere with cell functions and osmotic pressure; they can even directly inhibit the acid fermentation pathway (Palmqvist and Hahn-Hagerdal, 2000).

Acetic acid is an inhibitory substance that also exists in hydrolysates. It is formed by the hydrolysis of acetyl groups in hemicellulose and, to some extent, lignin (Klinke *et al.*, 2004). In the undissociated form, acetic acid can penetrate the cell membrane and inhibit product formation, disrupting the pH balance at high concentration, inhibiting cell growth or even killing cells (Klinke *et al.*, 2002). However, some strains can use acetic acid as a substrate to produce H_2_ (Matsumoto and Nishimura, 2007; Xu *et al.*, 2010).

Aromatics may arise in hydrolysates depending on the type of pretreatment applied and on the ratio of p-coumaryl alcohol, coniferyl, and sinapyl alcohol, the main lignin monomers. Pretreatment can transform lignin into a complex mixture of low-molecular-weight or "monomeric" phenolic compounds, especially by acid impregnation (Klinke *et al.*, 2004; Chen *et al.*, 2013). Phenolic compounds are well known for being toxic to microbial cells. They bear carboxyl, formyl, and hydroxyl groups, which increase the fluidity of the membrane and affect its permeability (Ren *et al.*, 2009).

In summary, the pretreatment of lignocellulosic material to use it as a substrate for producing H_2_ may generate fermentation inhibitors as well as other unusual substrates, such as pentose (xylose) and/or oligosaccharides (Maintinguer *et al.*, 2011; Quemeneur *et al.*, 2012b), which is a major drawback.

The use of xylose as a substrate appears to be less problematic than the presence of inhibitory compounds because xylose can be metabolized as illustrated in Figure 1. Indeed a series of H_2_-producing microorganisms, such as *Clostridium* spp. (Maintinguer *et al.*, 2011); *Enterobacter* spp. CN1 (Long *et al.*, 2010); and the thermophiles *Thermoanaerobacterium saccharolyticum* (Ren *et al.*, 2008; Shaw *et al.*, 2008), *Thermotoga neapolitana* DSM 4359 (Ngo *et al.*, 2012), *Caldicellulosiruptor saccharolyticus* (de Vrije *et al.*, 2009) and Thermoanaerobacterium thermosaccharolyticum (Khamtib and Reungsang, 2012), can consume and produce hydrogen from xylose. Ren *et al.* (2008) reported that *T. saccharolyticum* W16 can ferment a mixture of glucose and xylose with a H_2_ yield of up to 2.37 mol of H_2_/mol of substrate.

However, inhibitors such as furan derivatives and phenolic compounds negatively affect H_2_ production by mixed cultures. According to Quéméneur *et al.* (2012), furans exert a more negative effect than that induced by phenolic compounds. These authors found that *Clostridium beijerinckii* strains resisted these inhibitors better than other clostridial and non-clostridial bacteria did; therefore, *C. beijerinckii* is a promising microorganism for H_2_ production from lignocellulosic hydrolysates. Tai *et al.* (2010) observed that higher phenol concentrations (1 g/L) significantly inhibited *C. butyricum* metabolism. Nevertheless, no metabolic inhibition or co-degradation occurred at concentrations of approximately 0.6 g/L. Veeravalli *et al.* (2013) observed that furans affected fermentative H_2_ production by a mixed anaerobic culture. Furan levels of up to 1 g/L favored propionate and ethanol generation, decreasing H_2_ production.

In conclusion, the main limitation of using pretreated lignocellulosic materials in fermentative H_2_ production is the presence of these inhibitors.

## H_2_ Production From Non-Pretreated Lignocellulosic Materials

Because pretreatment processes are expensive and can produce inhibitory compounds, it would be beneficial to avoid pretreatment and directly convert lignocellulosic materials to H_2_ (Levin *et al.*, 2009; Raj *et al.*, 2012).

Only a few reports concerning the production of H_2_ from untreated lignocellulosic feedstocks exist in the literature (Ren *et al.*, 2009), and most of them involve thermophilic microorganisms. For example, *Clostridium thermocellum* ATCC 27405 and *C. saccharolyticus* DSM 8903 can hydrolyze cellulose and hemicellulose to produce H_2_ (Raj *et al.*, 2012).

C. saccharolyticus can produce H_2_ directly from mechanically comminuted switchgrass without any chemical or biological pretreatment (Talluri *et al.*, 2013).

Some authors have resorted to co-cultures that allow for the use of lignocellulosic materials as substrates. Wang *et al.* (2008) reported that a co-culture consisting of *Clostridium acetobutylicum* and *Ethanoigenens harbinense* effectively hydrolyzed cellulose and produced H_2_ from microcrystalline cellulose. Li and Liu (2012) developed a co-culture of *C. thermocellum* and *C. thermosaccharolyticum*, to improve hydrogen production via the thermophilic fermentation of cornstalk waste. The authors achieved a hydrogen yield of 68.2 mL of H_2_/g of cornstalk, 94.1% higher than the yield obtained using a monoculture of *C. thermocellum*.

Table 1 lists results for fermentative H_2_ production from lignocellulosic materials without any chemical pretreatment, the employed inocula, and the H_2_ yield obtained from these substrates. The results are presented as maximum assessed production yield, as indicated by the authors; when possible, we converted the data and expressed them as maximum calculated production yield (mmol of H_2_/g of substrate) for comparison. All the wastes included in Table 1 were milled before being assayed.

**Table 1:** Fermentative H_2_ production from lignocellulosic residues without pretreatment: employed inoculum and H_2_ yield obtained from these substrates.

Feedstock	Inoculum	T (°C)	Maximum assessed production yield^a^	Maximum calculated production yield (mmol H_2_/g of substrate)^b^	Reference
Cornstalk	C. thermocellum	55	61.4 mL of H_2_/g	2.28	Cheng and Liu, 2012
Cornstalk	anaerobic digester sludge	55	37.6 mL of H_2_/g	1.40	Cheng and Liu, 2012
Cornstalk	mixed microflora from rotted wood crumb	60	115.3 mL of H_2_/g	4.22	Cao *et al*., 2012
Cornstalk	*C. thermocellum*, *C. thermosaccharolyticum*	55	74.9 mL of H_2_/g	2.78	Li and Liu, 2012
Cornstalk	cow dung compost	36	3 mL of H_2_/g	0.12	Zhang *et al*., 2007
Mushroom cultivation waste	heated mixed cultures	55	0.73 mmol of H_2_/g	0.73	Lay *et al*., 2012
Grass (Reed canary)	H_2_-microbial enrichment culture	35	0.19 mmol of H_2_/g	0.19	Lakaniemi *et al*., 2011
Grass	mixed cultures enriched with *C. pasteurianum*	35	4.39 mL of H_2_/g	0.17	Cui and Shen, 2012
Grass (switchgrass)	*C. saccharolyticus* DSM 8903	65	11.2 mmol of H_2_/g	11.2	Talluri *et al*., 2013
Rice straw	*T. neapolitana*	75	2.3 mmol of H_2_/g	2.3	Nguyen *et al*., 2010
Rice straw	sewage sludge	55	21 mL of H_2_/g	0.78	Kim *et al*, 2013
Wheat straw	preheated anaerobic sludge	37	10.52 mL of H_2_/g VS^c^	0.41	Quemeneur *et al*., 2012 (a)
Wheat straw	*C. saccharolyticus*	70	44.7 mL of H_2_/g	1.59	Ivanova *et al*., 2009

a Maximum assessed production yields are the results presented by the authors.

b Maximum calculated production yields are results converted from authors' data determined according to the ideal gas equation considering a pressure of 1 atm and the absolute temperature used during H_2_ fermentation.

c VS: Volatile solids contained in the substrate.

The temperature clearly affected the fermentative H_2_ production yield from lignocellulosic residues. Most of the studies that used untreated lignocellulosic materials employed thermophilic conditions (10, n = 14) to provide yields approximately 75% higher than those obtained under mesophilic conditions. Although most studies employed a mixed culture as an inoculum, C. thermocellum and T. thermosaccharolyticum, previously known as *C. thermosaccharolyticum* were the thermophilic microorganisms most frequently employed to produce H_2_ from untreated feedstock.

The untreated raw materials presented in Table 1 afforded an average maximum calculated H_2_ production yield of 2.17 (± 1.84) mmol of H_2_/g of substrate; yields ranged from 0.12 to 11.2 mmol of H_2_/g of substrate. The only study on switchgrass furnished the highest yield − 11.2 mmol of H_2_/g of substrate (Talluri *et al.*, 2013). When we excluded this study from the calculations, the average H_2_ production yield from untreated lignocellulosic substrates decreased to 1.41 (± 1.02) mmol of H_2_/g, where the highest average yield observed was that obtained for cornstalk − 2.16 (± 1.17) mmol of H_2_/g.

## H_2_ Production From Pretreated Lignocellulosic Materials

Although some studies on the direct conversion of lignocellulosic materials to H_2_ exist, most microorganisms require pretreated lignocellulosic material as a substrate to produce biohydrogen. The degree of pretreatment depends on the nature of the raw material and on the inoculated organism(s) (Ren *et al.*, 2009).

Most pretreatment steps generate undesirable inhibitors, but they significantly enhance H_2_ production. Zhang *et al.* (2007) improved biohydrogen production from cornstalk after acidification and heat pretreatment. The authors achieved maximum cumulative H_2_ production of 150 mL of H_2_/g of VS after treating the substrate with 0.2% HCl; this production was 50 times higher than the value obtained without pretreatment. Cornstalks treated with NaOH (0.5%) furnished 57 mL of H_2_/g of VS, *i.e.*, 19-fold the initial value obtained for the raw material (3 mL of H_2_/g of VS) (Zhang *et al.*, 2007).

Table 2 summarizes literature results concerning the use of pretreated lignocellulosic wastes, the pretreatment type, the inoculum, and the H_2_ yield obtained from these substrates. The results shown in Table 2 refer to the maximum assessed production yield, as indicated by the authors; when possible, we converted the data and expressed them as maximum calculated production yield (mmol H_2_/g of substrate) for comparison.

**Table 2:** Fermentative H_2_ production from pretreated lignocellulosic residues, pretreatment type, inoculum, and H_2_ yield obtained from these substrates.

Feedstock	Pretreatment	Inoculum	T (°C)	Maximum assessed production yield^a^	Maximum calculated production yield (mmol H_2_/g of substrate)^b^	Reference
Beet pulp	pH 12 with NaOH for 30 min	anaerobic sludge	35	115.6 mL of H_2_/g of COD	-	Ozkan *et al*., 2011
Corn stalk	Lime loading of 0.10 g/g of biomass for 96 h	mixed microflora from rotted wood crumb	60	155.4 mL of H_2_/g of TVS	5.69	Cao *et al*., 2012
Cornstalk	*Phanerochaete chrysosporium*	*T. thermosaccharolyticum*	50	89.3 mL of H_2_/g	3.99	Zhao *et al*., 2013
	*Trichoderma viride*	*T. thermosaccharolyticum*	50	90.6 mL of H_2_/g	4.04	Zhao *et al*., 2013
Cornstalk	solid state enzymolysis	panda manure	36	205.5 mL of H_2_/g of TVS	8.11*	Xing *et al*., 2011
Cornstalk	H_2_SO_4_ 0.5% at 121°C for 60 min	microwave irradiated cow dung compost	36	144.3 mL of H_2_/g	6.44	Song *et al*., 2012
Cornstalk	NaOH at 120 °C for 20 min	anaerobic sludge	55	45.7 mL of H_2_/g	1.70	Cheng and Liu, 2012 (a)
Cornstalk	Fungal pretreatment	anaerobic sludge	55	54.1 mL of H_2_/g of VS	2.01*	Cheng and Liu, 2012 (b)
Cornstalk	Acidification 0.2% HCl	cow dung compost	36	149.69 mL of H_2_/g of TVS	5.90*	Zhang *et al*., 2007
Corn stover	1.2% H_2_SO_4_/2 h and steam explosion 200 °C for 1 min	dried sludge	35	184.71 mL of H_2_/10 g (18.47 mL/g)	0.73	Datar *et al*., 2007
Corn stover	Microwave assisted acid pretreatment (H_2_SO_4_ 0.3 N for 45 min)	anaerobic sludge	55	18.22 mL of H_2_/g	0.68	Liu and Cheng, 2010
Grass	4% HCl	anaerobic	35	72.21 mL of H_2_/g	2.86	Cui and Shen 2012
	0.5% NaOH	mixed bacteria	35	19.25 mL of H_2_/g	0.86	Cui and Shen 2012
Grass (Reed canary)	3% HCl solution for 90 min at 121 °C	H_2_-fermenting microbial enrichment culture	35	1.25 mmol of H_2_/g	1.25	Lakaniemi *et al*., 2011
Rapeseed stillage	Alkaline peroxide with steam treatment	digested manure	55	79 mL of H_2_/gVS	2.94*	Luo *et al*., 2011
Rapeseed cake	Alkaline peroxide with steam treatment	digested manure	55	24 mL of H_2_/gVS	0.89*	Luo *et al*., 2011
Rice straw	10% ammonia and 1.0% H_2_SO_4_	*T. neapolitana*	75	2.7 mmol of H_2_/g	2.70	Nguyen *et al*., 2010
Sugarcane bagasse	0.5% H_2_SO_4_ for 60 min at 121 °C	*C. butyricum*	37	1.73 mol of H_2_/mol sugar	-	Pattra *et al*., 2008
Sugarcane bagasse	H_2_SO_4_ at 1% for 60 min at 121 °C	preheated elephant dung	37	0.84 mol of H_2_/mol sugar	-	Fangkum and Reunsang, 2011
Sugarcane bagasse	H_2_SO_4_ at 1% for 60 min at 121 °C	*T. thermosaccharolyticum*	55	1.12 mol of H_2_/mol sugar	-	Saripan and Reungsang, 2013
Waste ground wheat	H_2_SO_4_, pH 3.0, 90 °C for 15 min	preheated anaerobic sludge	37	946.2 mL	-	Sagnak *et al*., 2011
Wheat straw	HCl pretreated	cow dung compost	36	68.1 mL of H_2_/g TVS	3.04*	Fan *et al*., 2006
Wheat straw	Hydrothermic 180 °C for 15 min	preheated anaerobic sludge	70	7.36 mmol of H_2_/g sugars	-	Kongjan *et al*., 2010

a Maximum assessed production yields are the results as presented by the authors.

b Maximum calculated production yields results converted from authors' data calculated according to the ideal gas equation considering a pressure of 1 atm and the absolute temperature used during H_2_ fermentation.

* Maximum calculated production yield/g of substrate calculated as mmol H_2_/g of total volatile solids (TVS) or volatile solids (VS) contained in the substrate.

Acid and base pretreatment have been the pretreatments most frequently employed to prepare lignocellulosic materials for biohydrogen production − 11 and 6 studies, respectively, from the 21 publications presented in Table 2 have been reported. Enzymatic and/or biological pretreatment represent 3 of the 21 studies shown in Table 2. Only one study involved the use of temperature alone.

As indicated by the maximum calculated production yield data presented in Table 2, the biological pretreatment afforded the highest average yield 4.54 (± 1.78) mmol of H_2_/g of substrate compared with the acid and basic pretreatment (2.94 ± 1.85 and 2.41 ± 1.52 mmol of H_2_/g of substrate, respectively). Therefore, pretreatment effectiveness depended on the feedstock and pretreatment conditions, such as acid or base concentration, exposure time, and temperature.

According to Table 2, the average H_2_ production yield from pretreated material was 3.17 (± 1.79), ranging from 0.68 to 8.11 mmol of H_2_/g of substrate for corn stover and cornstalk, respectively. Pretreated cornstalk furnished the highest average yield 4.74 (± 1.80) mmol of H_2_/g of substrate, which was approximately 2.2 times higher that yielded by untreated cornstalk (2.17 ± 1.84 mmol of H_2_/g of substrate, Table 1). Therefore, the pretreatment step enhances H_2_ production.

Most studies used a mixed culture of microorganisms previously enriched with H_2_-producing bacteria as an inoculum. The thermophilic *T. thermosaccharolyticum* was the pure culture most frequently employed in the studies using pretreated lignocellulosic wastes as substrates.

## H_2_ Production From Lignocellulosic Materials Hydrolysates

The structural changes that prehydrolysis (pretreatment) promotes in a lignocellulosic matrix positively affect the subsequent enzymatic hydrolysis of lignocellulosic materials, increasing the saccharification yield (Ren *et al.*, 2009). Several authors have used this strategy to increase the concentration of sugars in hydrolysates for H_2_ production (de Vrije *et al.*, 2009; Cui *et al.*, 2010; Luo *et al.*, 2011; Pan *et al.*, 2011; Monlau *et al.*, 2013b). Pan *et al.* (2011) pretreated cornstalk containing 81.7% TVS with dilute acid, *i.e.*, 1.5% H_2_SO_4_, at 121 °C for 60 min, followed by enzymatic hydrolysis at 52 °C, pH 4.8, with an enzyme loading of 9.4 IU/g, to obtain a total soluble sugar content of 562.1 ± 6.9 mg/g of TVS during the stages of hydrolysis. The maximum hydrogen yield from this hydrolysate using an anaerobic mixed culture was calculated in terms of grams of cornstalk (TVS) as 209.8 mL of H_2_/g of TVS.

Pretreatment followed by enzymatic hydrolysis is a very efficient method for saccharifying lignocellulosic substrates. However, depending on the type of substrate and pretreatment conditions employed, the hydrolysates could inhibit fermentative H_2_ production. Monlau *et al.* (2013b) verified that hydrolysates from sunflower stalks pretreated with dilute acid negatively affected H_2_-producing microflora. The dilute acid pretreatment condition that these authors employed (170 °C, 1 h, 4 g of HCl/100 g of TS) was highly efficient in hydrolyzing hemicellulosic material because approximately 3.14 g/L of xylose and only 0.28 g/L of glucose emerged in the slurry. In addition to the amount of xylose, other byproducts arose - formate (0.6 g/L) and acetate (0.81 g/L), and furan derivatives such as furfural (1.15 g/L) and HMF (0.13 g/L). In a batch system inoculated with mixed microflora, 15% of this hydrolysate completely inhibited H_2_ production.

In a long-term experiment, Arreola-Vargas *et al.* (2013) observed that partial replacement of a synthetic medium containing glucose and xylose with an acid and with an enzymatic hydrolysate of oat straw, in a continuous reactor, diminished H_2_ production. The acid hydrolysate consisted mainly of glucose 1.5 g/L and xylose 3.7 g/L as well as phenolic compounds, such as HMF (133.2 mg/L), furfural (0.6 mg/L), and vanillin (3.59 mg/L). The enzymatic hydrolysate contained 3.8 g/L of glucose and 1.3 g/L of xylose, but no HMF, furfural, or vanillin. Both hydrolysates were used to feed an anaerobic sequencing batch reactor by gradually substituting the glucose/xylose medium with the hydrolysates. The substitution of glucose/xylose by the acid hydrolysate disaggregated the granules and interrupted the process. On the other hand, the replacement of the glucose/xylose medium with the enzymatic hydrolysate without fermentation inhibitors elicited H_2_ production. However, the H_2_ yield and production rate decreased from 2 mol of H_2_/mol of sugar and 278 mL of H_2_/L.h to 0.81 mol of H_2_/mol of sugar and 29.6 mL H_2_/L.h, respectively, in going from the synthetic medium to the enzymatic hydrolysate (Arreola-Vargas *et al.*, 2013).

Simultaneous saccharification and fermentation (SSF) has been successfully conducted to produce H_2_ from pretreated or even untreated lignocellulosic substrates by adding hydrolytic enzyme(s) or by seeding hydrolytic enzymes produced in the same fermentation vessel. Thus, in this approach, no pretreatments or only mild conditions for pretreating substrates are necessary, diminishing the formation of fermentation inhibitors (see Figure 2) because most saccharification occurs simultaneously with the fermentation (Lakshmidevi and Muthukumar, 2010; Quemeneur *et al.*, 2012a; Zhao *et al.*, 2013). For example, Quemeneur *et al.* (2012a) used a mixed culture of microorganisms and evaluated the efficiency of exogenous enzyme addition during fermentative H_2_ production from wheat straw. The authors used two experimental designs: a one-stage system (direct enzyme addition) and a two-stage system (enzymatic hydrolysis prior to fermentation). H_2_ production from untreated wheat straw ranged from 5.18 to 10.52 mL of H_2_/g of *vs.* H_2_ production yields increased two-fold and ranged from 11.06 to 19.63 mL of H_2_/g of VS after the enzymatic treatment of the wheat straw. Direct addition of exogenous enzymes during one-stage dark fermentation was the best way to improve H_2_ production from lignocellulosic biomass.

Table 3 summarizes the lignocellulosic material hydrolysates used as substrates for fermentative H_2_ production, the pretreatment and enzymatic hydrolysis methods used, the source of inoculum or the microorganisms involved in the fermentation, and the process yields and/or rates. Results regarding H_2_ yields from hydrolysates are expressed in terms of mmol of H_2_/mmol of sugar or mmol of H_2_/g of substrate because it was not always possible to convert these units. In the last case, it was possible to compare data with the results of untreated and pretreated substrates (Table 1 and 2).

**Table 3:** Fermentative H_2_ production from hydrolysates of lignocellulosic substrates according to pretreatment type and enzymatic hydrolysis, inocula, yields, and maximum production rate obtained from these substrates.

Feedstock	Pretreatment/ hydrolysis	Inoculum	T (°C)	Maximum production yield (a, b, b*)	Maximum production rate (mmol of H_2_/L.h)	Reference
Conifer pulp	55%H_2_SO_4_ at 45 °C for 2 h, neutralized with Ca(OH)_2_	preheated anaerobic sludge	37	2.26^a^	nd	Nissilä *et al*., 2012
Corn stover	Delignification with 2% NaOH+hydrolysis with cellulase and xylanase	*T. thermosaccharolyticum*	60	nd	11.2	Ren *et al*., 2010
Cornstalk	Dilute acid+enzymatic hydrolysis	anaerobic mixed microflora	36	8.58^b^	nd	Pan *et al*., 2011
Cornstalk	Fungal hydrolysis by *Trichoderma viride*	*T. thermosaccharolyticum* W16	60	3.28^b^	nd	Zhao *et al*., 2013
Miscanthus crop	Alkaline pretreatment at 75 °C+enzymatic hydrolysis	*C. saccharolyticus*	70	2.9^a^	12.6	de Vrije *et al*., 2009
		*T. neapolitana*	70	3.4^a^	13.1	de Vrije *et al*., 2009
Oat straw	HCl at 2%+90 °C for 2 h	two anaerobic sludges, heated at 100 °C for 30 min.	30	2.9^a^	3.3	Arriaga *et al*., 2011
Poplar leaves	HCl at 4%+2% Viscozyme	anaerobic mixed bacteria	35	1.78^b^	nd	Cui *et al*., 2010
Rapeseed	Alkaline peroxide with steam treatment+celluclast and β-glucosidase	digested manure	55	3.38b*	nd	Luo *et al.*
Rice straw	Alkaline pretreatment+*Acinetobacter junii* F6-02 enzymes	*C. butyricum* CGS5	37	0.76^a^	1.05	Lo *et al*., 2010
Sugarcane bagasse	Pretreated with H_3_PO_4_+*Cellulomonas uda* enzymes	*C. butyricum* CGS5	37	1.08^a^	nd	Lo *et al*., 2011
Sugarcane bagasse	Alkaline and enzymatic hydrolysis with cellulase from *Pseudomonas sp*.	*C. pasteurianum*	37	0.96^a^	1.38	Cheng and Chang, 2011
Sugarcane bagasse	NaOH 0.1 mol/L at 100 °C for 2 h and hydrolysis with cellulase	preheated anaerobic sludge	35	13.4b*	0.28^c^	Chairattanamanokorn *et al*., 2009
Sunflower stalks	HCl 4 g at 170 °C for 1 h/100 gTS	preheated anaerobic sludge	35	2.04^a^	nd	Monlau *et al*., 2013 (b)
Sweet sorghum bagasse	Pretreatment with NaOH+cellulase	*C. saccharolyticus*	72	2.6^a^	10.2 – 10.6	Panagiotopoulos *et al*, 2010
Wheat straw	SSF (acid+enzymatic)	anaerobic sludge	36	5.56b*	nd	Nasirian *et al*., 2011
Wheat straw	Ozone and simultaneous enzymatic hydrolysis	preheated cow manure and pond sediment preheated	35	3.2^b^	nd	Wu *et al*., 2013
Wheat straw	SSF (*Trichoderma*+fermentation)		37	0.80b*	nd	Quemeneur *et al*., 2012 (a)
	SSF (acid+enzymatic saccharification prior to fermentation)	preheated anaerobic sludge	37	0.45b*	nd	Quemeneur *et al*., 2012 (a)

a Maximum production yield in terms of mmol of H_2_/mmol of sugar.

b Maximum production yield in terms of mmol of H_2_/g of substrate.

b* Maximum production yield in terms of mmol of H_2_/g of total volatile solids (TVS) or volatile solids (VS) contained in the substrate.

c Maximum production rate in terms of mmol of H_2_/h.g TVS.

nd: not determined.

According to Table 3 the H_2_ production yields from hydrolysates ranged from 0.45 to 13.39 mmol of H_2_/g of substrate, for wheat straw and sugarcane bagasse, respectively.

Cornstalk is the most often studied lignocellulosic substrate for H_2_ production. The average yield using a cornstalk hydrolysate for biohydrogen production is 5.93 mmol of H_2_/g of substrate, which is approximately 270% and 25% higher than that afforded by the untreated (2.17 mmol of H_2_/g of substrate) and pretreated cornstalk (4.74 mmol of H_2_/g of substrate), respectively. The results demonstrated that after pretreatment and/or hydrolysis, this substrate is potentially applicable in biohydrogen production.

Although sugarcane bagasse afforded the highest yield − 13.39 mmol of H_2_/g of TVS; this figure represents the results obtained in only one study (Chairattanamanokorn *et al.*, 2009). The average H_2_ production yield per mol of sugar of pretreated bagasse was 1.23 mol of H_2_/mol of glucose (Table 2); for the hydrolysates, this yield dropped to 1.12 (Table 3), demonstrating that H_2_ production from hydrolysates of this substrate was slightly lower.

Excluding the work of Chairattanamanokorn *et al.* (2009) with sugarcane bagasse, the average H_2_ production yield with sugarcane bagasse hydrolysates (Table 3) was 3.78 ± 1.92 mmol of H_2_/g, 20% higher compared with the average yields of pretreated substrates. However, this average H_2_ production yield was lower than that of biologically pretreated substrates, 4.54 ± 1.78 mmol of H_2_/g. These results demonstrate the importance of avoiding the presence of inhibitors originating from chemical pretreatment methods.

## Conclusions and Perspectives

Based on this review, converting agroindustrial lignocellulosic substrates to H_2_ by fermentative microorganisms is a feasible solution for producing H_2_ sustainably. However, additional research into the pretreatment of lignocellulosic wastes for biohydrogen production is desirable to improve the yield and make the process economically viable. Efforts to control the formation (or removal) of toxic compounds (such as furan derivatives, phenolics, and organic acids, formed during the chemical pretreatment) are necessary because these could clearly inhibit H_2_ fermentation. Biological pretreatment methods afford higher H_2_ yields from lignocellulosic materials because they do not produce inhibitors.

The development of microbial strains or consortia resistant to inhibitors remains an important research area. Moreover, the discovery of novel H_2_-producing microorganisms able to use lignocellulosic derivatives is associated with different environmental conditions, particularly high temperatures.

Currently, results have shown that corn stalk submitted to a pretreatment step and/or hydrolysis furnishes a higher average yield of biohydrogen production than that afforded by other agroindustrial lignocellulosic substrates. Exploring other microorganisms and optimizing the pretreatment and hydrolysis conditions can make the use of this substrate and other agroindustrial residues a sustainable way to produce clean H_2_.
